# Artificial Photosynthesis of Glycolaldehyde and Syngas from Plastic Feedstocks via Boron‐Functionalized Nickel Species on CdS

**DOI:** 10.1002/anie.202517025

**Published:** 2025-09-28

**Authors:** Shuai Zhang, Xintong Gao, Bingquan Xia, Ashley Slattery, Jingrun Ran, Shi‐Zhang Qiao

**Affiliations:** ^1^ School of Chemical Engineering The University of Adelaide Adelaide SA 5005 Australia; ^2^ Key Laboratory for Green Chemical Process of Ministry of Education School of Chemistry and Environmental Engineering Wuhan Institute of Technology Wuhan Hubei 430074 China

**Keywords:** Bio‐inspired structure, Photocatalysis, Plastic upcycling, Proton‐coupled electron transform, Selective redox

## Abstract

Glycolaldehyde is an important intermediate in the synthesis of pharmaceuticals and biodegradable plastics. Artificial photosynthesis of glycolaldehyde from plastic waste provides a sustainable approach for waste recycling and solar energy utilization. However, the inertness of plastic substrates and unselective photoredox make it challenging to generate valuable aldehydes or other market‐demanded products. Here we demonstrate co‐production of glycolaldehyde and syngas from the photoreforming of polyethylene terephthalate via an electron–proton cascade redox using a boron‐functionalized nickel species modified CdS photocatalyst (Ni*_n_* B@Ni‐BO*_x_*/CdS) under ambient conditions. We confirm the surface specie as a nickel boride@nickel borate core‐shell nanoarchitecture with dual functions, serving as a reduction cocatalyst and facilitating electron–proton cascade transfer. The feature boosts charge separation and reactant molecule activation for an efficient cooperative redox. The optimized photocatalyst exhibits a glycolaldehyde generation of 1068.3 µmol g_cat_^−1^ h^−1^ with a selectivity of 66.3%, as well as a syngas generation of 3232.2 µmol g_cat_^−1^ h^−1^ with a tuneable H_2_/CO ratio. The finding demonstrates the solar‐driven synthesis of value‐added and multifunctional products from plastic waste as a sustainable and economically promising strategy.

## Graphical abstract

Inspired by enzyme cofactors, a CdS‐supported nickel boride@nickel borate core‐shell nanoarchitectured photocatalyst was developed, featuring closely integrated electron/proton acceptors and catalytic sites. This “all‐in‐one” photocatalyst boosted charge separation and facilitated electron–proton cascade transfer, enabling selective co‐synthesis of high‐value glycolaldehyde and versatile syngas from plastic feedstocks under ambient conditions.

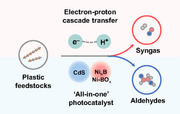

## Introduction

The chemical industry accounts for 30% of total industrial energy use, relying primarily on fossil fuels as both chemical feedstocks and energy sources, resulting in a significant carbon footprint worldwide.^[^ ^1^, ^2^, ^3^ ^]^ The increasing emphasis on sustainable and carbon‐neutral development necessitates a transition in material and energy usage within the chemical sector, leveraging recovered waste feedstocks and renewable energy instead of fossil fuels to produce valuable industrial products.^[^ ^4^, ^5^ ^]^ For instance, plastic waste accumulated in landfills poses serious environmental concerns, but it can be recycled and reused as a carbon feedstock for chemical synthesis.^[^ ^6^, ^7^, ^8^ ^]^ Chemical recycling is an industrially important approach for converting plastic waste into monomers and valued chemicals, but the harsh reaction conditions required lead to significant energy consumption and waste generation. Photocatalysis is a sustainable technology that utilizes renewable solar energy to drive chemical reactions for artificial photosynthesis under mild conditions.^[^ ^9^, ^10^, ^11^, ^12^, ^13^, ^14^ ^]^ Therefore, solar‐powered photocatalysis offers a promising approach to mitigating plastic waste and producing valuable products toward a sustainable circular economy.^[^ ^15^, ^16^, ^17^ ^]^

Glycolaldehyde is a simple sugar molecule containing both alcohol and aldehyde functionalities, serving as a valuable platform molecule for the synthesis of pharmaceuticals and biodegradable polymers with high market demand.^[^ ^18^ ^]^ Another important platform molecule, syngas, primarily composed of H_2_ and CO, is widely used to produce versatile fuels and chemicals. However, the industrial production of both valued platform chemicals relies on energy‐intensive processes using fossil‐based feedstocks at high reaction temperature.^[^ ^18^, ^19^ ^]^ Given the key role of photo‐induced charge carriers in boosting reaction efficiency and regulating reaction selectivity,^[^ ^20^, ^21^ ^]^ the photocatalytic conversion of plastic waste presents an appealing alternative for the sustainable synthesis of glycolaldehyde and syngas. Polyethylene terephthalate (PET) is the most widely produced polyester, yet its recycling rate remains below 20%.^[^ ^22^ ^]^ Developing waste PET as a synthetic feedstock is therefore crucial, as it helps mitigate nonbiodegradable plastic accumulation while leveraging its ester functional groups to synthesize valuable oxygenates. The reported products from the photoreforming of PET derivatives are limited to H_2_ and simple organic acids (e.g., formic acid), most of which have a lower market value than PET itself (∼1.1 US$ per kg).^[^ ^5^, ^22^, ^23^ ^]^ The synthesis of more functional platform molecules (e.g., syngas) and higher‐value chemicals (such as glycolaldehyde, ∼9.0 US$ per kg)^[^ ^24^ ^]^ from plastic waste via photocatalysis is economically advantageous but remains unexplored. To achieve this, it is necessary to create an appropriate reaction microenvironment that i) facilitates the separation and transfer of photo‐induced charge carriers; ii) boosts H_2_ evolution reaction (HER); and iii) regulates the reactivity of oxidative species to activate C─H bond toward C─C bond cleavage without overoxidizing aldehyde intermediates. Addressing these challenges requires the rational design of photocatalysts.

**Figure 1:**
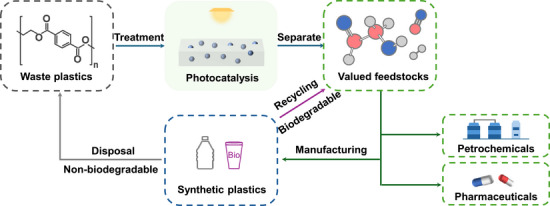
Conceptual illustration of a sustainable plastic cycle. Artificial photosynthesis of glycolaldehyde and syngas through the photocatalytic upcycling of waste plastics, with these products serving as potential feedstocks for manufacturing. Ball‐and‐stick model: red = carbon, blue = oxygen, and grey = hydrogen.

Here we present an “all‐in‐one” catalyst design by incorporating a nickel boride@nickel borate core‐shell nanoarchitecture on CdS (Ni*_n_* B@Ni‐BO*_x_*/CdS) to boost electron–proton cascade redox for the concurrent production of glycolaldehyde and syngas from PET substrates. We evidence that the core nickel boride extracts photo‐induced electrons from CdS for reduction, while the holes on CdS synergize with the shell nickel borate for dehydrogenation and proton transfer, boosting substrate oxidation. Inspired by enzyme cofactors in photosynthesis, the “all‐in‐one” design integrates the electron acceptor (core nickel boride and CdS) and the proton acceptor (shell nickel borate) in close proximity, enabling electron–proton cascade transfer. This favors the charge separation within the photocatalyst and boosts the activation of C─H bond in substrate molecules by holes with mild oxidation ability, thus contributing to efficient and selective photoredox. We confirm that Ni*_n_* B@Ni‐BO*_x_*/CdS exhibits a glycolaldehyde production of 1068.3 µmol g_cat_^−1^ h^−1^ with a selectivity of 66.3%, significantly outperforming pristine CdS (50 µmol g_cat_^−1^ h^−1^ and 15.1%), as well as stable glycolaldehyde generation over 65 h of photoreforming. The yield and composition of syngas can be readily tuned by controlling the content and structure of the Ni*_n_* B@Ni‐BO*_x_* species. The glycolaldehyde and syngas generated are of significant industrial value and could potentially be applied in plastic manufacturing, contributing to a circular economy (Figure 1). Our findings demonstrate a sustainable strategy for the co‐production of versatile platform molecules and high‐value chemicals from plastic waste through the rational design of photocatalysts.

## Results and Discussion

### Synthesis and Structure Characterization of Ni*_n_* B@Ni‐BO*_x_*/CdS

Photoreforming involves the water reduction half‐reaction and the organic oxidation half‐reaction using waste substrates.^[^ ^5^ ^]^ The oxidation of plastic derivatives is initiated by oxidative species through C─H bond scission and hydrogen abstraction, determining overall reaction efficiency and product selectivity.^[^ ^25^, ^26^ ^]^ Proton‐coupled electron transfer plays an important role in natural photosynthesis and photocatalytic organic synthesis,^[^ ^27^, ^28^, ^29^ ^]^ with the potential to boost C─H bond activation and photoredox. Inspired by the positioning of cofactors in enzymes,^[^ ^28^ ^]^ designing a structure that associates a proton acceptor with a redox catalyst could facilitate electron–proton cascade transfer. Given these considerations, CdS was synthesized as a support photocatalyst because of its excellent light absorption and mild oxidizing ability, which helps prevent the overoxidation of intermediates and products.^[^ ^25^ ^]^ Nickel boride exhibits metalloid conductivity and special electronic structure, which is favorable for boosting charge separation/transfer within photocatalysts.^[^ ^30^, ^31^, ^32^ ^]^ Nickel borate, with borate “base” sites that function similarly to oxyanions,^[^ ^33^, ^34^ ^]^ is expected to accept protons. Therefore, we developed a nickel boride@nickel borate core‐shell nanoarchitecture‐modified CdS via a chemical reduction method. The integration of a proton acceptor and a redox photocatalyst within a single structure renders the Ni*_n_* B@Ni‐BO*_x_*/CdS composite a promising platform for photoreforming.

**Figure 2:**
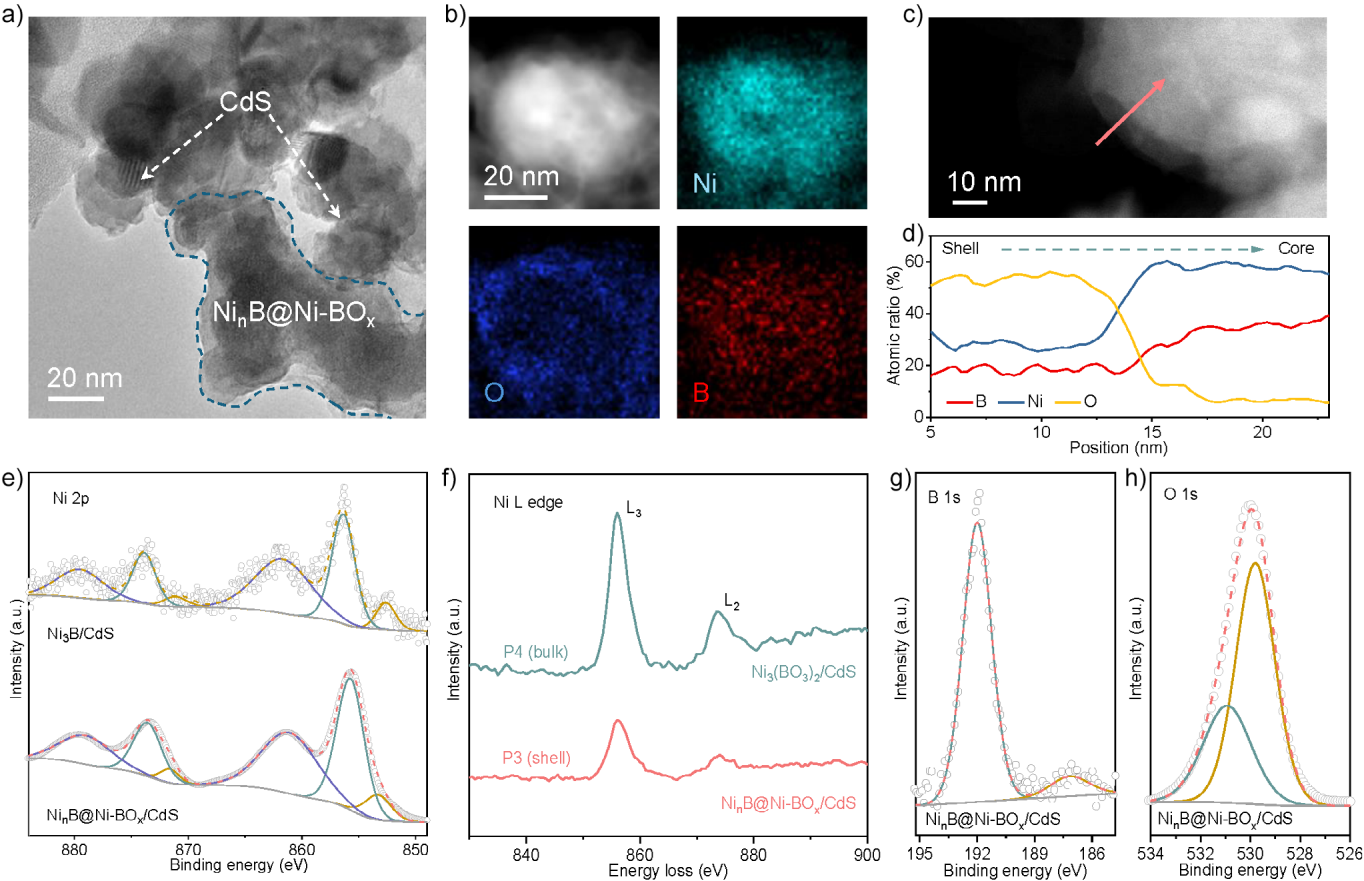
Structure characterization for Ni*_n_* B@Ni‐BO*_x_*/CdS. a) HRTEM image and b) EDS mapping for Ni*_n_* B@Ni‐BO*_x_*/CdS. c) HAADF‐STEM image for a selected area of Ni*_n_* B@Ni‐BO*_x_*/CdS. d) Atomic percent composition based on the EELS line scan analysis in c). e) Ni 2p XPS spectra for Ni*_n_* B@Ni‐BO*_x_*/CdS and Ni_3_B/CdS. f) Ni L‐edge EELS spectra for Ni*_n_* B@Ni‐BO*_x_*/CdS and Ni_3_(BO_3_)_2_/CdS. g) B 1s and h) O 1s XPS spectra for Ni*_n_* B@Ni‐BO*_x_*/CdS.

High‐resolution transmission electron microscopy (HRTEM) imaging of Ni*_n_* B@Ni‐BO*_x_*/CdS confirmed that Ni*_n_* B@Ni‐BO*_x_*, in the form of interconnected nanoparticles, was combined with CdS nanoparticles (Figure 2a). High‐angle annular dark‐field scanning transmission electron microscopy (HAADF‐STEM) imaging of Ni*_n_* B@Ni‐BO*_x_*/CdS exhibited crystalline CdS with a lattice spacing of 0.32 nm, corresponding to the (101) plane of CdS,^[^ ^34^ ^]^ as well as amorphous Ni*_n_* B@Ni‐BO*_x_* with a core‐shell structure (Figure S1). X‐ray diffraction (XRD) patterns for CdS and Ni*_n_* B@Ni‐BO*_x_*/CdS exhibited the diffraction peaks for wurtzite CdS with no detectable Ni‐ and B‐related features (Figure S2), consistent with HAADF‐STEM findings. The measured specific surface area of Ni*_n_* B@Ni‐BO*_x_*/CdS was 45.7 m^2^ g^−1^ (Table S1). Energy‐dispersive spectroscopy (EDS) mappings exhibited a uniform distribution of Ni and B throughout the core‐shell structure, with O enriched in the outer shell (Figure 2b). This is consistent with the electron energy loss spectroscopy (EELS) elemental analysis, where CdS nanoparticles and surface O‐rich core‐shell nanostructure constitute the Ni*_n_* B@Ni‐BO*_x_*/CdS composite (Figure S3), corresponding to HRTEM and HAADF‐STEM results. The EELS line scan of the shell‐to‐core region exhibited that the average atomic contents of Ni, B, and O in the O‐rich shell were 28%, 18%, and 52%, respectively, while those of Ni and B in the O‐deficient core were 59% and 32% (Figure 2c,d). The amorphous core‐shell nanostructure is therefore determined to consist of nickel boride (Ni*_n_* B) as the core nanoparticle and nickel borate (Ni–BO*_x_*) as the outer layer, based on elemental distribution, atomic ratio, and independent reports.^[^ ^35^, ^36^ ^]^ During reduction, Ni*_n_* B formed on CdS and facilitated the hydrolysis of sodium borohydride to borate ions. Following interacting with nickel cations, Ni–BO*_x_* grew on the surface of active Ni*_n_* B, leading to a Ni*_n_* B@Ni‐BO*_x_* core‐shell structure.^[^ ^35^ ^]^ Additionally, Ni_3_B and Ni_3_(BO_3_)_2_ were synthesized and combined with CdS nanoparticles to form Ni_3_B/CdS and Ni_3_(BO_3_)_2_/CdS as reference samples.^[^ ^36^ ^]^ The successful synthesis of these two was confirmed via HAADF‐STEM and XRD analyses (Figure S4).

To confirm the elemental chemical states of Ni*_n_* B@Ni‐BO*_x_*/CdS, X‐ray photoelectron spectroscopy (XPS) and EELS were performed. As shown in Figure 2e, the Ni 2p signal of Ni*_n_* B@Ni‐BO*_x_*/CdS comprises Ni^0^ (853.1 eV) and Ni^2+^ (855.8 eV),^[^ ^35^, ^37^, ^38^ ^]^ evidencing that the surface Ni*_n_* B@Ni‐BO*_x_* species exist in a mixed valence state of Ni^0^ and Ni^2+^. The Ni^0^ peak, which is similar to that of Ni_3_B/CdS, originates from the inner Ni*_n_* B nanoparticles. The slight difference in Ni^0^ binding energy between Ni*_n_* B@Ni‐BO*_x_*/CdS and Ni_3_B/CdS is most likely because of the distinct outer Ni^2+^ species in the two samples and the corresponding interaction with the core Ni*_n_* B. The Ni^0^/Ni^2+^ ratios for Ni*_n_* B@Ni‐BO*_x_*/CdS and Ni_3_B/CdS were 0.18 and 0.21, respectively. Therefore, Ni*_n_* B@Ni‐BO*_x_*/CdS has a lower amount of metallic Ni^0^ and a higher average Ni oxidation state compared with Ni_3_B/CdS. Figure S5 exhibited the EELS spectra for the core area (P1) of Ni*_n_* B@Ni‐BO*_x_*/CdS and the bulk area (P2) of Ni_3_B/CdS. The Ni L‐edge spectral features of Ni*_n_* B@Ni‐BO*_x_*/CdS and Ni_3_B/CdS are similar (Figure S5c), indicating comparable Ni oxidation states in the selected regions of both samples.^[^ ^35^ ^]^ The L_3_/L_2_ area ratio for Ni*_n_* B@Ni‐BO*_x_*/CdS was computed to be 4.12. Additionally, Ni*_n_* B@Ni‐BO*_x_*/CdS and Ni_3_B/CdS presented a single broad peak in the B K‐edge spectra and no detectable O K‐edge characteristic signal (Figure S5d,e). This indicates an O‐deficient structure with similar B coordination environments in both the core Ni*_n_* B and bulk Ni_3_B.^[^ ^39^, ^40^ ^]^ EELS findings confirmed the core structure of Ni*_n_* B@Ni‐BO*_x_* species as a metal boride, consistent with XPS and HAADF‐STEM findings.

The shell structure of Ni*_n_* B@Ni‐BO*_x_*/CdS was confirmed via EELS spectra from the shell region (P3) of Ni*_n_* B@Ni‐BO*_x_*/CdS and the bulk area (P4) of Ni_3_(BO_3_)_2_/CdS (Figure S6a,b). For Ni L‐edge spectra, Figure 2f exhibited two sharp L_3_ and L_2_ peaks with similar L_3_/L_2_ area ratios of 3.61 for Ni*_n_* B@Ni‐BO*_x_*/CdS and 3.64 for Ni_3_(BO_3_)_2_/CdS. The finding confirms that the Ni oxidation state in the outer shell is close to that of Ni_3_(BO_3_)_2_ in the analyzed regions and primarily exists as Ni^2+^, which is reinforced by the XPS result (Figure S6c). The lower Ni L_3_/L_2_ area ratio obtained in the shell (3.61) compared with the core (4.12) confirms a higher Ni oxidation state in the shell and a lower Ni oxidation state in the core, corresponding to nickel borate and nickel boride, respectively. The shell of Ni*_n_* B@Ni‐BO*_x_*/CdS and bulk Ni_3_(BO_3_)_2_/CdS exhibited π* and σ* signals in B K‐edge EELS spectra, evidencing B coordination environment in the shell distinct from that in the core (Figures S5d and 6d). This is confirmed by the B 1s XPS spectra for Ni*_n_* B@Ni‐BO*_x_*/CdS (Figure 2g). The peak at around 187.2 eV is attributed to B signal in nickel boride, whilst the other at around 192 eV corresponds to B─O bonding in nickel borate.^[^ ^35^, ^36^ ^]^ Figure 2h presented the O 1s XPS spectra for Ni*_n_* B@Ni‐BO*_x_*/CdS, where two peaks at 529.8 and 530.9 eV were attributed to B─O─Ni and B─O bindings, respectively.^[^ ^35^, ^41^ ^]^ This is consistent with the O K‐edge EELS spectra for Ni*_n_* B@Ni‐BO*_x_*/CdS and Ni_3_(BO_3_)_2_/CdS (Figure S6e). The shell structure of Ni*_n_* B@Ni‐BO*_x_* species was determined to be a nickel borate based on EELS, XPS, and HAADF‐STEM findings. It is therefore concluded that the Ni*_n_* B@Ni‐BO*_x_* specie in Ni*_n_* B@Ni‐BO*_x_*/CdS consists of a nickel boride (i.e., Ni*_n_* B) core and a nickel borate (i.e., Ni‐BO*_x_*) shell.

### Photocatalytic Performance

The photocatalysts, including CdS, various Ni‐modified CdS and boron‐functionalized transition metal‐decorated CdS, were evaluated for photoreforming of ethylene glycol (EG) and the PET substrate solution under xenon lamp irradiation. PET substrate solution, primarily consisting of EG, was obtained through PET hydrolysis followed by acidification. EG, as the main reactant in PET photoreforming, was initially selected as the model substrate for photocatalyst screening and mechanistic study. Following 5 h of irradiation, the reduction product H_2_ and oxidation products, primarily including glycolaldehyde, acetic acid, and CO, were detected on pristine CdS (Figure S7). CdS exhibited low H_2_ generation of 34.5 µmol g_cat_^−1^ h^−1^ compared with oxidation products generation (420.5 µmol g_cat_^−1^ h^−1^), indicating its capacity to catalyze substrate oxidation but with limited HER activity. However, the accumulation of electrons could induce side reactions (e.g., self‐corrosion of CdS), compromising photocatalyst stability. The photocatalytic HER activity was significantly boosted following the introduction of Ni*_n_* B@Ni‐BO*_x_* species, resulting in a 76‐fold increase in H_2_ generation on Ni*_n_* B@Ni‐BO*_x_*/CdS (2614.4 µmol g_cat_^−1^ h^−1^) compared with pristine CdS (Figure S7). Increased oxidation products were observed on Ni*_n_* B@Ni‐BO*_x_*/CdS, confirming the important role of the Ni*_n_* B@Ni‐BO*_x_* species in boosting redox activity. This dual enhancement promotes efficient utilization of photo‐induced electrons and holes, boosting the stability of CdS photocatalysts. Among the oxidation products, glycolaldehyde was significantly produced and became the major product, along with increased CO generation and newly formed formaldehyde (Figure S7). This finding indicates that glycolaldehyde likely serves as an intermediate product in the formation of CO and formaldehyde. The generation of acetic acid on CdS (280 µmol g_cat_^−1^ h^−1^) and Ni*_n_* B@Ni‐BO*_x_*/CdS (283.3 µmol g_cat_^−1^ h^−1^) was similar, evidencing that the acetic acid formation is primarily dependent on the intrinsic activity of CdS. A small amount of CO_2_ by‐product (41.7 µmol g_cat_^−1^ h^−1^) was also detected on Ni*_n_* B@Ni‐BO*_x_*/CdS. Similar product distributions and yields were observed on both CdS and treated CdS, evidencing that the reactivity of CdS was unaffected under chemical reduction conditions (Figure S7). Based on product analysis, H_2_ and glycolaldehyde were the primary reduction and oxidation products of photoreforming on Ni*_n_* B@Ni‐BO*_x_*/CdS, respectively. Control experiments confirmed that no products were detectable in the absence of the photocatalyst, light, and reaction substrate (Figure S7). No detectable CO was produced in photocatalytic experiments using CO_2_ and H_2_O as the starting reactants. This finding confirms that CO originates from EG substrate conversion rather than from CO_2_ reduction. It was concluded that the detected products originated from the reaction substrate.

**Figure 3:**
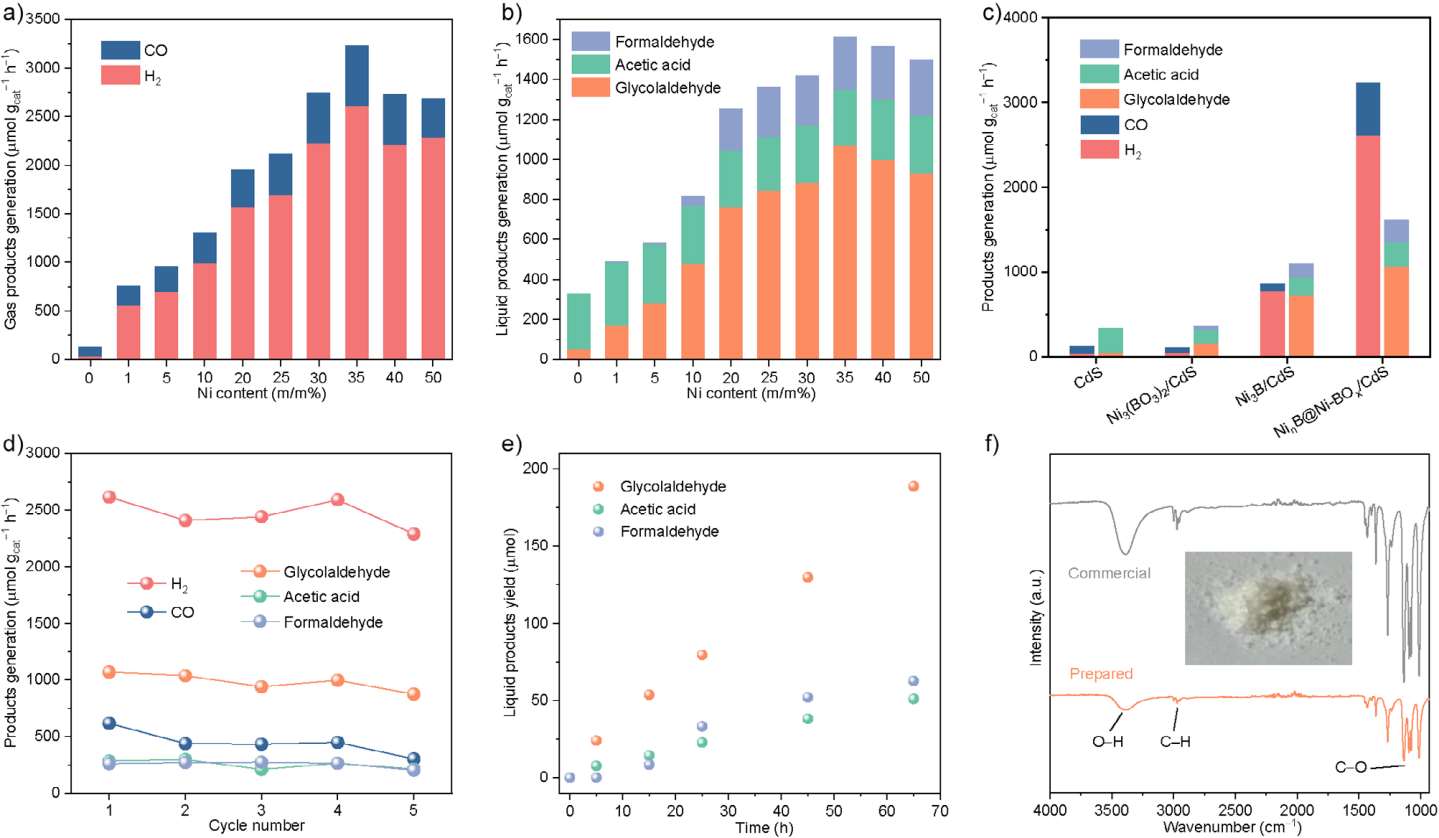
Photoreforming of EG and PET substrates on various CdS photocatalysts. Generation of a) gaseous products and b) liquid products on pristine CdS and Ni*_n_* B@Ni‐BO*_x_*/CdS with varying Ni contents following 5 h of EG photoreforming. c) Generation of products on pristine CdS, Ni_3_B/CdS, Ni_3_(BO_3_)_2_/CdS and Ni*_n_* B@Ni‐BO*_x_*/CdS in EG photoreforming. d) Cyclic measurements for Ni*_n_* B@Ni‐BO*_x_*/CdS with 5 h per cycle in EG photoreforming. e) Time‐dependent generation of liquid products from photoreforming of PET substrates over Ni*_n_* B@Ni‐BO*_x_*/CdS. f) FTIR spectra for prepared and commercial glycolaldehyde in dimer form. The inset shows a graphic photograph of the prepared product.

The photocatalytic activity of Ni*_n_* B@Ni‐BO*_x_*/CdS exhibited a volcano‐type trend with Ni content (Figure 3a,b). The greatest H_2_ and oxidation product yields were obtained on Ni*_n_* B@Ni‐BO*_x_*/CdS with a theoretical Ni content of 35 m/m%. The actual Ni content in Ni*_n_* B@Ni‐BO*_x_*/CdS was determined to be 11.4 m/m% (Table S1). The gaseous oxidation product CO is readily separated from the liquid oxidation product and forms an important feedstock syngas with the reduction product H_2_. Therefore, the gaseous and liquid products were classified and analyzed. As shown in Figure 3a, optimized Ni*_n_* B@Ni‐BO*_x_*/CdS exhibited an H_2_ generation of 2614.4 µmol g_cat_^−1^ h^−1^ and a CO generation of 617.8 µmol g_cat_^−1^ h^−1^. The H_2_/CO molar ratio can be tuned within the range of 0.4–5.6 by adjusting the Ni content, which is favorable for a variety of industrial applications. In the aqueous phase, glycolaldehyde is the major product, along with the formaldehyde and acetic acid generated. The greatest glycolaldehyde generation was 1068.3 µmol g_cat_^−1^ h^−1^, with a selectivity of 66.3% (Figure 3b). The glycolaldehyde yield of Ni*_n_* B@Ni‐BO*_x_*/CdS with varying Ni contents was presented in Table S2. The reaction substrate and liquid products were identified and quantified via ^1^H nuclear magnetic resonance (NMR) and high‐performance liquid chromatography with reference to calibration curves (Figures S8 and S9). Additionally, commonly used transition metals, including Fe, Co, Cu, and Mn, were incorporated into CdS via a similar synthetic method for photoreforming. As shown in Figure S10, Ni‐modified CdS, i.e., Ni*_n_* B@Ni‐BO*_x_*/CdS, exhibited superior photocatalytic performance in both reduction and oxidation product generation compared with other transition metal‐modified CdS. The finding highlights the excellent catalytic activity of the formed Ni*_n_* B@Ni‐BO*_x_* species. The Ni*_n_* B@Ni‐BO*_x_* specie was confirmed to consist of a nickel boride core and a nickel borate shell via a range of the characterizations. To determine the role of two components, photoreforming measurements were conducted using Ni_3_B/CdS, Ni_3_(BO_3_)_2_/CdS and Ni*_n_* B@Ni‐BO*_x_*/CdS with comparable Ni contents. As shown in Figure 3c, Ni_3_(BO_3_)_2_/CdS exhibited low photocatalytic activity, with a product yield comparable to pristine CdS. Ni_3_B enhanced the photocatalytic activity of CdS, as evidenced by the increased generation of both reduction and oxidation products on Ni_3_B/CdS. However, the overall performance of Ni_3_B/CdS was inferior to that of Ni*_n_* B@Ni‐BO*_x_*/CdS. These findings confirmed nickel boride as the active component, while the synergistic effect between nickel boride and nickel borate in the Ni*_n_* B@Ni‐BO*_x_* species significantly boosted the photoreforming performance.

The photocatalytic activity of Ni*_n_* B@Ni‐BO*_x_*/CdS in product generation was retained across five cyclic tests (Figure 3d). There was no evident change in the morphology, composition, and chemical state of Ni*_n_* B@Ni‐BO*_x_*/CdS following the photoreforming (Figure S11). These findings confirm the excellent stability of the Ni*_n_* B@Ni‐BO*_x_*/CdS photocatalyst. It is of practical importance to generate valuable chemicals via long‐term and stable photoreforming of PET substrates. Figure 3e exhibited that the amount of glycolaldehyde generated increased almost linearly with illumination over 65 h, leading to an accumulated yield of 188 µmol. The ratio of the amount of glycolaldehyde generated to its theoretical molar yield (derived from the EG content in the PET substrate solution) was calculated to be 36.5%. Following the separation of photocatalysts from the reaction solution, the product was collected from several batches through concentration, extraction, and crystallization. During dehydration, concentrated glycolaldehyde likely undergoes a self‐condensation reaction, resulting in the formation of a more stable dimeric form.^[^ ^42^ ^]^ The final product was confirmed by comparison with commercial samples using Fourier transform infrared spectroscopy (FTIR) and Raman spectra (Figures 3f and S12). Ni*_n_* B@Ni‐BO*_x_*/CdS exhibited excellent photocatalytic activity per mass of photocatalysts and substrates, comparable to representative systems reported for photocatalytic plastic conversion (Table S3). The efficient and selective production of high‐value glycolaldehyde and versatile syngas highlights the potential economic advantage of our photoreforming system.

### Photocatalytic Mechanistic Study

**Figure 4:**
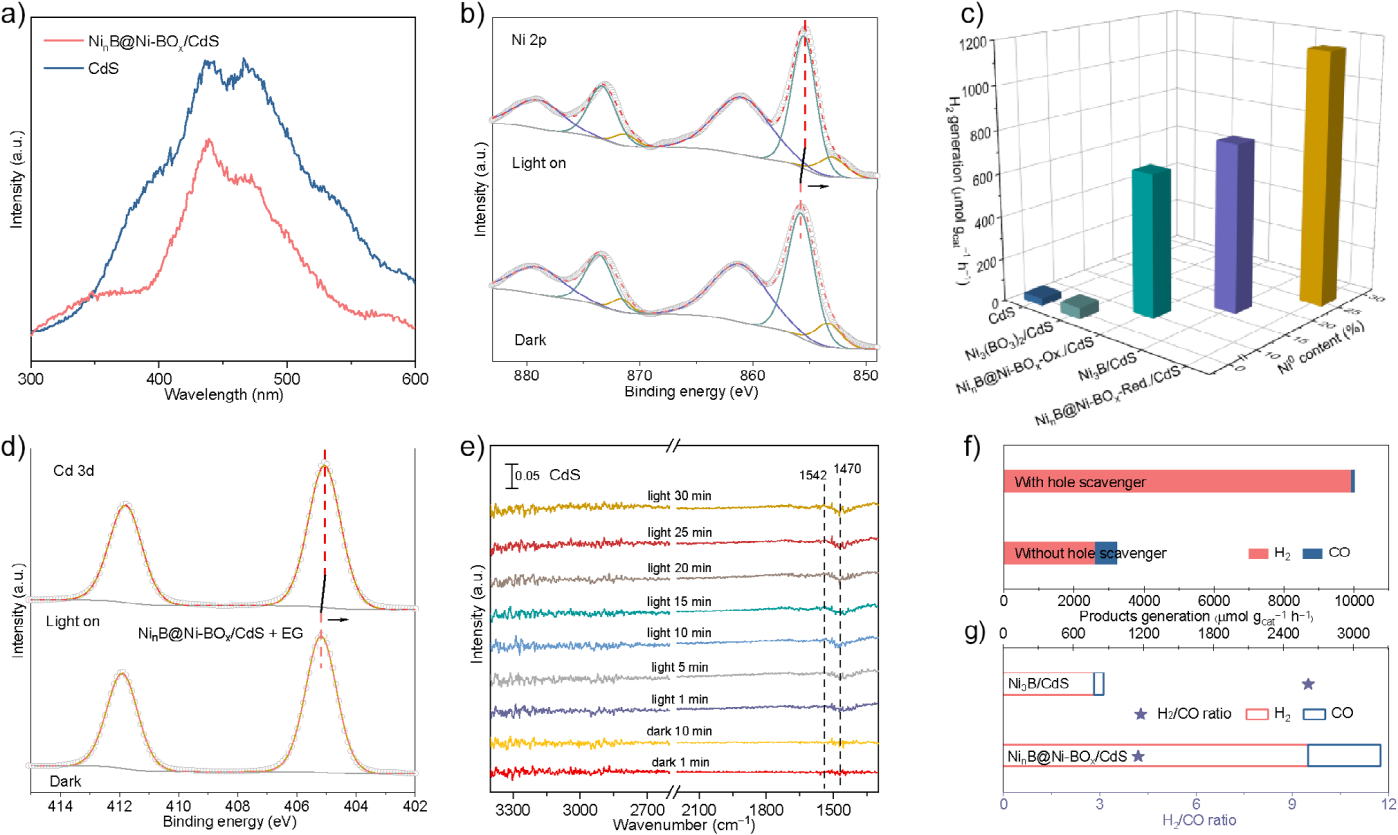
Charge transfer and surface reaction on photocatalysts. a) Steady PL spectra for CdS and Ni*_n_* B@Ni‐BO*_x_*/CdS. b) Ni 2p XPS spectra for Ni*_n_* B@Ni‐BO*_x_*/CdS in dark and under illumination. c) H_2_ generation on CdS and differing Ni‐modified CdS photocatalysts in EG photoreforming. d) In situ Cd 3d XPS spectra for Ni*_n_* B@Ni‐BO*_x_*/CdS with EG substrate and illumination. e) In situ DRIFTS spectra for EG substrate conversion on CdS under light irradiation. f) Generation of products on Ni*_n_* B@Ni‐BO*_x_*/CdS with and without hole capturing agent. g) H_2_/CO generation (top axis) and H_2_/CO ratio (bottom axis) for Ni_3_B/CdS and Ni*_n_* B@Ni‐BO*_x_*/CdS in EG photoreforming.

The basis for the boosted photocatalytic activity for Ni*_n_* B@Ni‐BO*_x_*/CdS was evaluated. According to UV–vis diffuse reflectance spectroscopy, Ni*_n_* B@Ni‐BO*_x_*/CdS presented enhanced light absorption in the range of ∼505–800 nm compared with CdS (Figure S13). However, the Ni*_n_* B@Ni‐BO*_x_*‐boosted light absorption did not significantly contribute to the photocatalytic activity of Ni*_n_* B@Ni‐BO*_x_*/CdS, because no H_2_ was detected under 630 nm light irradiation. The steady photoluminescence (PL) spectra for CdS and Ni*_n_* B@Ni‐BO*_x_*/CdS were presented as Figure 4a. Ni*_n_* B@Ni‐BO*_x_*/CdS exhibited reduced fluorescence emission compared with pristine CdS, evidencing that Ni*_n_* B@Ni‐BO*_x_* species suppressed the recombination of photo‐induced electrons and holes.^[^ ^43^ ^]^ Photo‐irradiated XPS was performed to determine the charge transfer pathway. As shown in Figures 4b and S14a, the Ni 2p main peak of Ni*_n_* B@Ni‐BO*_x_*/CdS shifts by 0.4 eV toward lower binding energy under light irradiation, accompanied by negatively shifted binding energy in B 1s, evidencing electron migration from CdS to Ni*_n_* B@Ni‐BO*_x_* species. The reduced charge recombination is mostly likely because of the boosted charge transfer to Ni*_n_* B@Ni‐BO*_x_* species, consistent with PL spectra. A positive shift was exhibited in the Cd 3d XPS spectra for Ni*_n_* B@Ni‐BO*_x_*/CdS following illumination (Figure S14b). These findings confirm that Ni*_n_* B@Ni‐BO*_x_* species extract electrons from CdS for the reduction reaction, while the photo‐induced holes are localized on CdS to initiate substrate oxidation.

Given the electron accumulation on surface Ni*_n_* B@Ni‐BO*_x_* species, nickel boride, featuring Ni in a reduced state, was confirmed to act as the HER cocatalyst because of the greater H_2_ generation on Ni_3_B/CdS compared with Ni_3_(BO_3_)_2_/CdS, Figure 3c. To determine the role of the Ni*_n_* B@Ni‐BO*_x_* structure in HER, Ni*_n_* B@Ni‐BO*_x_*/CdS catalysts with varying contents of reduced Ni were prepared and assessed via photoreforming experiments. Ni*_n_* B@Ni‐BO*_x_*‐Red./CdS and Ni*_n_* B@Ni‐BO*_x_*‐Ox./CdS were obtained via the partial reduction and oxidation of Ni*_n_* B@Ni‐BO*_x_*/CdS, respectively. As shown in Figures S15a and 16a, both samples maintained the nickel boride@nickel borate core‐shell structure. Ni*_n_* B@Ni‐BO*_x_*‐Red./CdS featured a nickel boride‐enriched core with a high content of reduced Ni, whereas Ni*_n_* B@Ni‐BO*_x_*‐Ox./CdS exhibits a partially oxidized core with a lower Ni° content compared with Ni*_n_* B@Ni‐BO*_x_*/CdS and Ni*_n_* B@Ni‐BO*_x_*‐Red./CdS. These findings were confirmed via HAADF‐STEM, EELS, XPS, and X‐ray absorption spectra (Figures S15 and S16). The Ni° content in the photocatalysts was positively correlated with HER activity. As shown in Figure 4c, the H_2_ generation was in the order Ni*_n_* B@Ni‐BO*_x_*‐Red./CdS > Ni_3_B/CdS > Ni*_n_* B@Ni‐BO*_x_*‐Ox./CdS > Ni_3_(BO_3_)_2_/CdS. This reinforces the important role of nickel boride in boosting HER, while excluding direct contributions from the core‐shell structure. Given the inertness of nickel borate toward HER, nickel borate is most likely to affect the oxidation and works synergistically with the nickel boride cocatalyst and CdS, thereby contributing to an excellent overall redox performance.

Following electron transfer to the Ni*_n_* B@Ni‐BO*_x_* species, the photo‐induced holes localized on CdS were involved in the substrate oxidation. This is confirmed via photo‐irradiated XPS in the presence of EG substrate. Figure 4d showed a negative shift in the Cd 3d binding energy of Ni*_n_* B@Ni‐BO*_x_*/CdS following illumination, which was most likely because of electron transfer between CdS component and EG reactant (i.e., an electron donor). The evolution of reactants and products on the CdS surface was established via in situ diffuse reflectance infrared Fourier transform spectroscopy (DRIFTS). Figure 4e presented the DRIFTS spectra for CdS in the presence of EG substrate, where weak infrared signals emerged upon illumination. The peak at 1470 cm^−1^, attributed to *δ*(C─H) bending in molecular EG,^[^ ^44^ ^]^ gradually decreased with illumination. This evidenced the consumption of EG via C─H bond dissociation on CdS. A vibrational band (1542 cm^−1^) of the carboxylic group was detected on the surface of CdS,^[^ ^8^, ^17^ ^]^ indicating the formation of carboxylic products, e.g., acetic acid. This was confirmed in the photocatalytic performance of CdS (Figure S7). Based on the valence band (VB) spectrum of CdS (Figure S17a), photo‐induced holes are not sufficiently oxidative to generate hydroxyl radicals and are therefore the primary active species for substrate oxidation, consistent with independent reports.^[^ ^23^ ^]^ In situ XPS and DRIFTS findings confirmed that CdS catalyzed substrate oxidation via hole‐induced C─H bond activation and electron transfer. This was reinforced via control experiment in which CO generation from substrate oxidation on Ni*_n_* B@Ni‐BO*_x_*/CdS was significantly reduced following quenching of photo‐induced holes (Figure 4f). However, H_2_ generation was boosted with the addition of hole scavengers because of improved charge separation and metal boride cocatalysts.

Compared with C_2_ products, CO was formed by multihole oxidation of substrate via C─C bond cleavage. Because of the inert C─C bond, this process is typically initiated by C─H bond activation and hydrogen abstraction in organic synthesis.^[^ ^45^ ^]^ As shown in Figure 4g, Ni*_n_* B@Ni‐BO*_x_*/CdS exhibited higher CO and H_2_ generation and a lower H_2_/CO ratio compared with Ni_3_B/CdS, indicating its excellent oxidation activity and hole selectivity toward CO formation.^[^ ^46^ ^]^ Given the similar product distributions (Figure 3c), hole‐induced C─H activation dominates the substrate oxidation in both Ni*_n_* B@Ni‐BO*_x_*/CdS and Ni_3_B/CdS. XPS VB spectra presented similar VB edge potentials for CdS and Ni*_n_* B@Ni‐BO*_x_*/CdS (Figure S17), evidencing that the oxidizing ability of holes on CdS is not the reason for the difference in photocatalytic activity. Therefore, the boosted CO generation on Ni*_n_* B@Ni‐BO*_x_*/CdS most likely originates from the role of the shell nickel borate in facilitating hydrogen abstraction. The synergy between Ni*_n_* B@Ni‐BO*_x_* species and CdS significantly boosts substrate oxidation via C─H/C─C bond activation and efficient HER, contributing to excellent redox activity.

**Figure 5:**
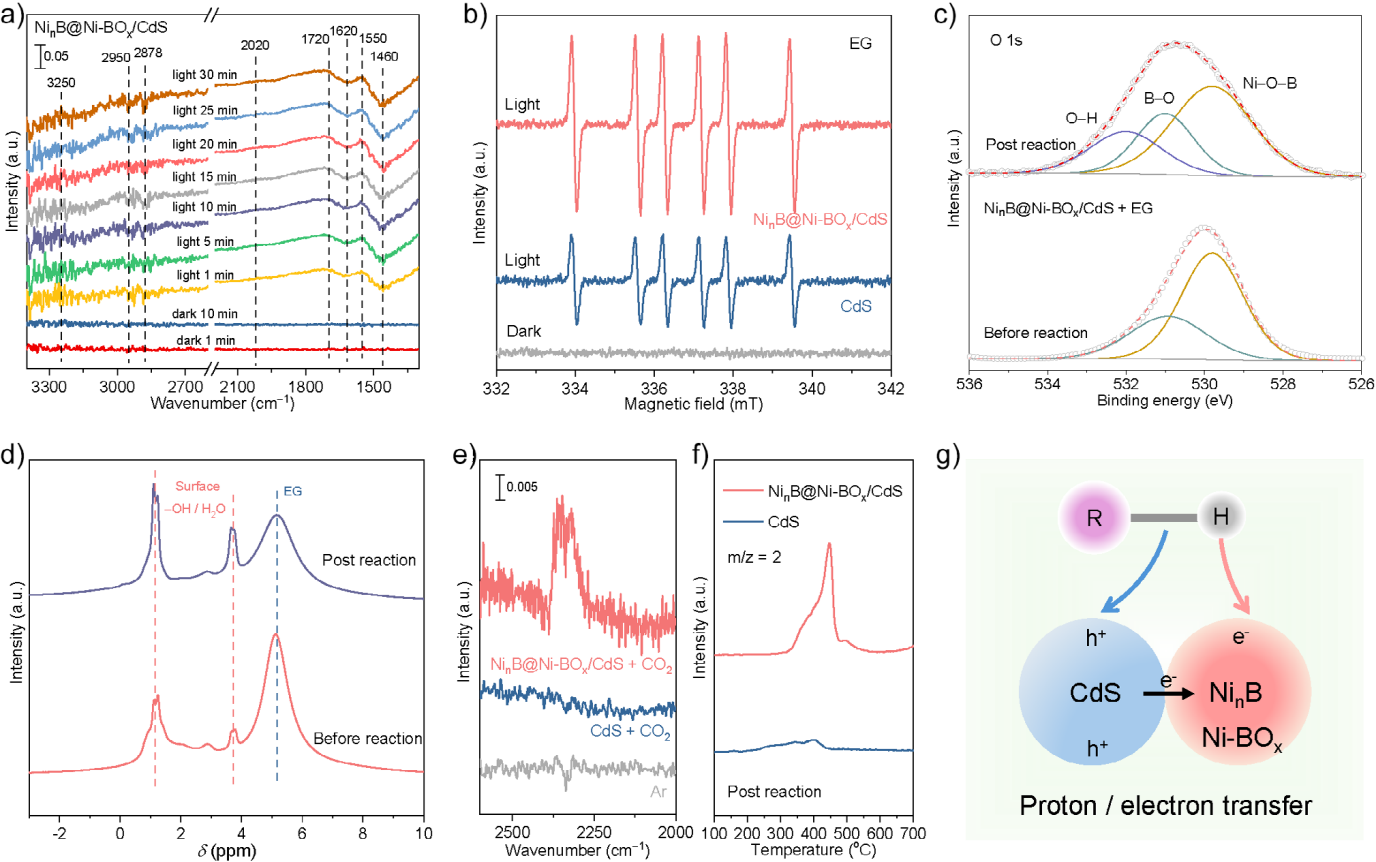
Characterization of photocatalytic substrate oxidation. a) In situ DRIFTS spectra for EG substrate conversion on Ni*_n_* B@Ni‐BO*_x_*/CdS under light irradiation. b) In situ EPR spectra for CdS and Ni*_n_* B@Ni‐BO*_x_*/CdS with EG substrate and illumination. c) O 1s XPS and d) solid‐state NMR spectra for Ni*_n_* B@Ni‐BO*_x_*/CdS with EG substrate before and following photoreaction. e) CO_2_ adsorption DRIFTS spectra for CdS and Ni*_n_* B@Ni‐BO*_x_*/CdS. f) TPD‐MS spectra for CdS and Ni*_n_* B@Ni‐BO*_x_*/CdS following photoreaction. g) Schematic for electron–proton cascade transfer in substrate activation on Ni*_n_* B@Ni‐BO*_x_*/CdS.

The role of Ni*_n_* B@Ni‐BO*_x_* species in substrate oxidation was confirmed via in situ spectroscopic characterizations. Figure 5a showed the DRIFTS spectra for the conversion of EG substrate on Ni*_n_* B@Ni‐BO*_x_*/CdS. Under light irradiation, the C─H bending (1460 cm^−1^) and stretching vibrations (2950/2878 cm^−1^) associated with the EG molecule were significantly decreased compared with those on CdS.^[^ ^17^ ^]^ This finding confirmed that Ni*_n_* B@Ni‐BO*_x_*/CdS boosted the dissociation of molecular EG via C─H bond activation. A band at 1720 cm^−1^, attributed to C═O stretching vibration of aldehyde species,^[^ ^17^, ^34^ ^]^ was apparent on Ni*_n_* B@Ni‐BO*_x_*/CdS (Figure 5a). These surface aldehydes corresponded to the formation of glycolaldehyde and formaldehyde products during the photoreaction. The band at 1620 cm^−1^ most likely corresponded to the O─H─O bending of adsorbed water,^[^ ^47^ ^]^ which was affected by the surface adsorption of EG substrate. No distinct characteristic absorption was observed in the control experiment (without adding EG), confirming that the detected intermediate and product signals originated from substrate conversion (Figure S18). These absorption features in DRIFTS spectra were also confirmed by adsorption experiments using varying concentrations of EG, glycolaldehyde, and acetic acid (Figure S19a–c). Additionally, a CO signal at 2020 cm^−1^ was detected on Ni*_n_* B@Ni‐BO*_x_*/CdS,^[^ ^8^ ^]^ originating from EG oxidation via C─C bond scission (Figures 5a and S19d). The finding confirms the role of Ni*_n_* B@Ni‐BO*_x_* species in boosting C─C bond activation, consistent with photocatalytic performance (Figure 4g).

To determine the evolution of surface species, photo‐irradiated XPS was performed in the presence of EG substrate (Figure S19e). The C 1s XPS spectra exhibited characteristic signals at 284.8 eV (C─H/C─C) and 286.3 eV (C─O),^[^ ^17^ ^]^ evidencing the adsorption of EG molecules on the Ni*_n_* B@Ni‐BO*_x_*/CdS surface. These two signals were significantly reduced following illumination, confirming the consumption of EG substrate. Electron paramagnetic resonance (EPR) experiments were conducted to examine the potential reaction intermediates generated by the photocatalysts. As shown in Figure 5b, a signal of carbon‐centered radical was detected on both CdS and Ni*_n_* B@Ni‐BO*_x_*/CdS following illumination.^[^ ^48^, ^49^, ^50^ ^]^ This radical most likely originated from the activation and hydrogen abstraction of the C─H bond during EG oxidation, providing a favorable pathway for subsequent C─C bond scission.^[^ ^25^, ^45^ ^]^ Ni*_n_* B@Ni‐BO*_x_*/CdS exhibited a stronger carbon‐centered radical signal than CdS, evidencing more efficient generation of the key reaction intermediate. This finding confirms the importance of Ni*_n_* B@Ni‐BO*_x_* species in boosting substrate oxidation via C─H/C─C bond activation, which is consistent with in situ DRIFTS and XPS data.

Electron–proton transfer is commonly involved in photocatalytic reactions.^[^ ^29^, ^45^ ^]^ In addition to electron transfer, hydrogen abstraction and transfer on the surface were also established. Figure 5c presented the O 1s XPS spectra for Ni*_n_* B@Ni‐BO*_x_*/CdS before and post reaction. An additional peak at 532 eV was apparent on postreaction sample, corresponding to surface hydroxyl formed during the photoreaction.^[^ ^51^, ^52^ ^]^ The hydroxyl species likely originated from hydrogen extraction from the hydrogen donor (i.e., substrate molecules) activated by holes, followed by transfer to the photocatalyst surface.^[^ ^53^ ^]^ This finding was confirmed via in situ DRIFTS (Figure 5a). An OH stretching peak appeared at a high wavenumber (3250 cm^‒1^) on the Ni*_n_* B@Ni‐BO*_x_*/CdS in the presence of EG substrate following illumination,^[^ ^54^, ^55^ ^]^ whereas no similar absorbance was observed on Ni_3_B/CdS (Figure S20). This indicates that the shell nickel borate boosts hydrogen extraction from EG and hydrogen transfer to the catalyst surface, forming adsorbed hydrogen as hydroxyl groups. Solid‐state NMR was performed to explore the hydrogen extraction and adsorption (Figure 5d). The broad peak at 5.2 ppm for Ni*_n_* B@Ni‐BO*_x_*/CdS was assigned to the hydroxyl hydrogen of the EG substrate.^[^ ^56^ ^]^ A decrease in this peak following photoreaction confirmed the EG conversion. Two main resonance peaks at 3.7 and 1.1 ppm corresponded to water molecules and surface hydroxyl groups, respectively.^[^ ^53^ ^]^ The increased hydroxyl peak was apparent on the postreaction Ni*_n_* B@Ni‐BO*_x_*/CdS, evidencing the formation of surface hydroxyls during the photoreaction. This most likely originated from the interaction between the abstracted hydrogen and the shell nickel borate.

Oxygen atoms in borate ions, featuring lone pairs of electrons, act as surface basic sites that accept hydrogen atoms from donors,^[^ ^52^, ^57^ ^]^ forming surface hydroxyl groups. Figure 5e exhibited the DRIFTS spectra for CO_2_ adsorption. A distinct CO_2_ vibrational absorbance at 2350 cm^−1^ was visible on Ni*_n_* B@Ni‐BO*_x_*/CdS, whilst no corresponding signal was detected on CdS. This is most likely because of CO_2_ adsorption boosted by the surface basic sites of Ni*_n_* B@Ni‐BO*_x_*/CdS.^[^ ^34^, ^53^ ^]^ Surface‐adsorbed hydrogen induced by Ni*_n_* B@Ni‐BO*_x_* species was also established via temperature‐programmed desorption‐mass spectrometry (TPD‐MS) measurements of post‐reaction samples (Figure 5f). Compared with CdS, Ni*_n_* B@Ni‐BO*_x_*/CdS exhibited a stronger H_2_ (*m*/*z* = 2) desorption signal, likely originating from surface‐adsorbed hydrogen atoms that were quenched by hydrogen acceptors under reaction conditions. This finding evidenced a greater amount of adsorbed hydrogen on the photocatalyst, confirming that Ni*_n_* B@Ni‐BO*_x_* species boosted hydrogen extraction and adsorption. Therefore, an electron–proton transfer pathway in EG substrate oxidation was resolved (Figure 5g). The C─H bond in EG molecules was activated by photo‐induced holes on CdS, followed by electron transfer to CdS. The shell nickel borate in the Ni*_n_* B@Ni‐BO*_x_* acted as a proton acceptor, abstracting hydrogen released from the substrate and forming surface‐adsorbed hydrogen via basic sites and photo‐induced electrons. This electron–proton cascade transfer favored efficient C─H bond activation and boosted the conversion of EG substrates.

**Figure 6:**
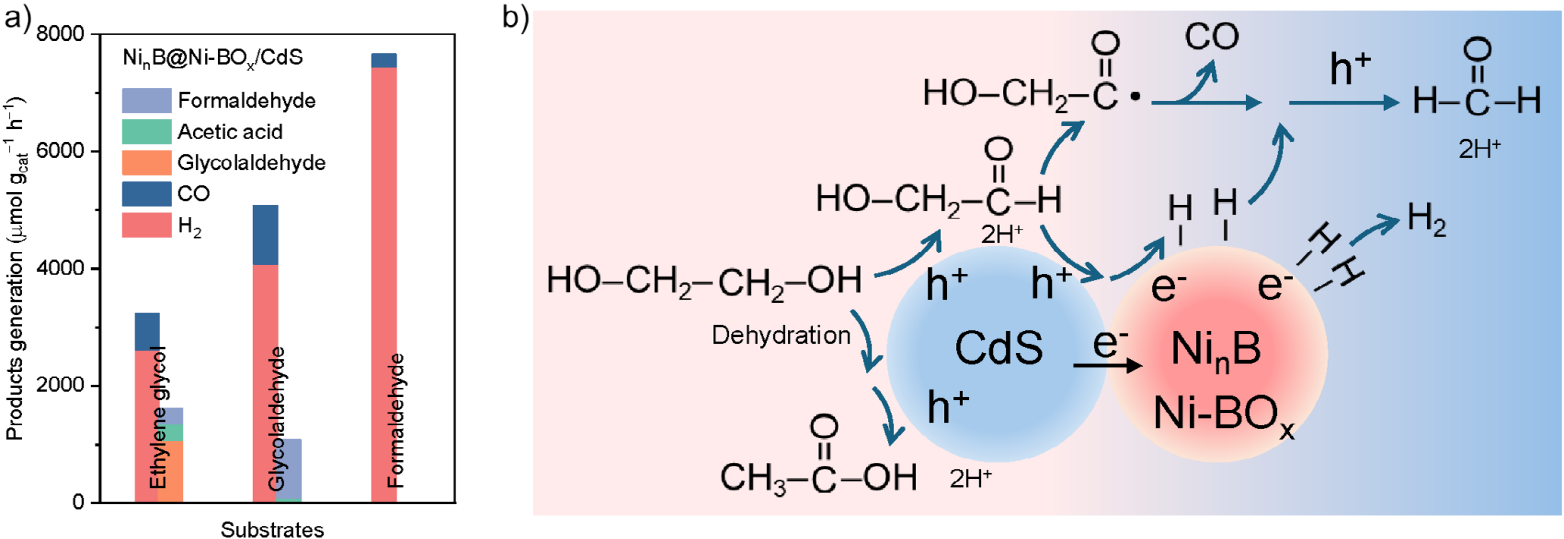
Mechanistic understanding. a) Generation of products from photoreforming of different substrates using Ni*_n_* B@Ni‐BO*_x_*/CdS photocatalysts. b) Hypothesized reaction routes for photoreforming of PET‐derived EG on Ni*_n_* B@Ni‐BO*_x_*/CdS.

EG substrate was activated via an electron–proton cascade transfer and subsequently oxidized by photo‐induced holes into glycolaldehyde as the major oxidation product. Glycolaldehyde acted as a potential intermediate for the formation of formaldehyde and CO,^[^ ^26^ ^]^ as confirmed by a comparative experiment. Figure 6a showed the photocatalytic performance of Ni*_n_* B@Ni‐BO*_x_*/CdS using EG, glycolaldehyde, and formaldehyde as starting substrates. Photoreforming of glycolaldehyde produced more H_2_ and CO than that of EG while maintaining a similar H_2_/CO ratio and yielding formaldehyde as the liquid product. However, CO generation from formaldehyde oxidation was significantly less than that from the other substrates. Therefore, glycolaldehyde is the main intermediate in the transformation to formaldehyde and CO. To determine the subsequent reaction pathway, in situ DRIFTS experiment was carried out on Ni*_n_* B@Ni‐BO*_x_*/CdS using glycolaldehyde substrate (Figure S21). The C─H vibration of the aldehyde group at 2724 cm^−1^ decreased with illumination, indicating that the oxidation of glycolaldehyde was initiated by hole‐induced dehydrogenation.^[^ ^26^, ^34^ ^]^ This process was also likely boosted by an electron–proton cascade transfer on Ni*_n_* B@Ni‐BO*_x_*/CdS. Subsequently, the decarbonylation of aldehyde groups generated CO,^[^ ^25^ ^]^ as evidenced by the significant decrease in the carbonyl absorption band at 1720 cm^−1^ following irradiation. The remaining methyl group, formed via C─C cleavage, was continuously oxidized by photo‐induced holes into a small amount of formaldehyde. This was confirmed via DRIFTS spectra, in which C─H‐related vibrations (2947, 2890, and 1438 cm^−1^) were significantly decreased upon illumination. Another byproduct, acetic acid, likely originates from light‐induced dehydration of EG followed by hole oxidation.^[^ ^58^ ^]^ The formation of acetic acid is primarily dependent on the intrinsic dehydration activity of CdS, resulting in similar yields on both CdS and Ni*_n_* B@Ni‐BO*_x_*/CdS (Figure S7).

Hypothesized reaction routes for photoreforming of the PET‐derived EG substrate by Ni*_n_* B@Ni‐BO*_x_*/CdS are presented as Figure 6b. Upon light irradiation, electron‐hole pairs are generated within Ni*_n_* B@Ni‐BO*_x_*/CdS. Electrons are excited to the conduction band of CdS and subsequently transfer to the Ni*_n_* B@Ni‐BO*_x_* species, leaving holes on the VB of CdS. The core nickel boride extracts electrons for efficient charge separation and catalyzes HER with electrons. Holes on CdS oxidize the C─H bond of the substrate molecule, while the shell nickel borate boosts hydrogen abstraction and transfer. The resulting electron–proton cascade transfer facilitates C─H bond activation and generates carbon‐centered radical intermediates, which are subsequently oxidized by holes to yield glycolaldehyde. Glycolaldehyde is oxidized by photo‐induced holes via dehydrogenation, followed by decarbonylation to generate CO. The other intermediate derived from C─C cleavage, most likely methanol,^[^ ^25^ ^]^ is eventually oxidized into formaldehyde. Acetic acid is generated through a minor pathway involving dehydration and hole oxidation. Based on the quantified redox products in photoreforming and the corresponding electron/hole consumption (Figure 3a,b), the hypothesized reaction mechanism follows the charge balance and the redox stoichiometry. From a mechanistic perspective, several strategies can be considered to further boost photoreforming performance. These include regulating the composition and structure of Ni*_n_* B@Ni‐BO*_x_* species to enhance substrate activation and H_2_ evolution, engineering reactors to enable efficient product‐catalyst separation and suppress overoxidation, and developing CdS photocatalysts with high surface areas to increase active species loading and improve charge separation/transfer.

## Conclusion

Concurrent synthesis of glycolaldehyde and syngas from PET photoreforming has been demonstrated using a modified CdS photocatalyst with multifunctional nickel boride@nickel borate core‐shell nanoarchitecture. The “all‐in‐one” structure design, which integrates the electron acceptor (core nickel boride and CdS) with the proton acceptor (shell nickel borate), facilitates charge separation and electron–proton cascade transfer for efficient substrate activation, thereby enabling the selective photoreforming of PET feedstocks into valued‐added products. The optimized Ni*_n_* B@Ni‐BO*_x_*/CdS presented a glycolaldehyde generation of 1068.3 µmol g_cat_^−1^ h^−1^ with a liquid‐phase selectivity of 66.3%, along with a syngas generation of 3232.2 µmol g_cat_^−1^ h^−1^ with an H_2_/CO ratio tuneable by the content and composition of surface Ni*_n_* B@Ni‐BO*_x_* species. The electron–proton cascade transfer, enabled by an enzyme‐mimicking structural design, boosts the conversion of plastic substrates through C─H/C─C bond activation and presents a promising approach for efficient and selective organic synthesis via photocatalysis. Findings demonstrate a sustainable photocatalytic strategy for synthesizing market‐demanded and versatile products from plastic waste, providing promising alternative to energy‐intensive and carbon‐emitting chemical manufacturing and waste treatment.

## Conflict of Interests

The authors declare no conflict of interest.

## Supporting information



Supporting information

## Data Availability

The data that support the findings of this study are available from the corresponding author upon reasonable request.
