# Supplementary material for: Artificial Photosynthesis of Glycolaldehyde and Syngas from Plastic Feedstocks via Boron‐Functionalized Nickel Species on CdS
Source: Angew Chem Int Ed Engl. 2025 Sep 28;64(48):e202517025. doi: 10.1002/anie.202517025 (PMC12643351; doi:10.1002/anie.202517025)
Supplement: Supplementary file 1 — Supporting information [file ANIE-64-e202517025-s001.docx]

Supporting Information

Artificial Photosynthesis of Glycolaldehyde and Syngas from Plastic Feedstocks *via* Boron-Functionalized Nickel Species on CdS

Shuai Zhang,^[a]^ Xintong Gao,^[a]^ Bingquan Xia,^[b]^ Ashley Slattery,^[a]^ Jingrun Ran,*^[a]^ and Shi-Zhang Qiao*^[a]^

[a] School of Chemical Engineering, The University of Adelaide, SA 5005, Australia.

[b] Key Laboratory for Green Chemical Process of Ministry of Education, School of Chemistry and Environmental Engineering, Wuhan Institute of Technology, Wuhan, Hubei 430074, China.

^*^Corresponding E-mail: jingrun.ran@adelaide.edu.au; s.qiao@adelaide.edu.au

**Experimental Section**

**Photocatalysts preparation.** CdS was prepared *via* a hydrothermal method. 5 mL of 0.55 M sodium sulfide aqueous solution was added dropwise to 22 mL of 0.13 M cadmium nitrate aqueous solution under stirring, and the mixture was stirred for an additional 1 h. The resulting solution was transferred to a 50 mL Teflon-lined autoclave and heated at 180^o^C for 12 h. Following cooling to room temperature (RT), the products were washed several times with ultrapure water and ethanol, and dried at 60^o^C for 12 h. A wet chemical reduction method was used to synthesize the boron-functionalized Ni species modified CdS photocatalysts. Typically, 20 mg of CdS was dispersed in 4.5 mL of ultrapure water under sonication. A selected amount of 77 mM nickel chloride aqueous solution was added to the CdS dispersion, followed by stirring for 30 min. Subsequently, 0.4 M of freshly prepared sodium borohydride aqueous solution was added stepwise under rigorous stirring, and the resultant solution was stirred for an additional 1 h. The obtained products were washed 4 times with ultra-pure water and dried at 40^o^C under vacuum for 12 h. The prepared photocatalyst is denoted Ni_n_B@Ni-BO_x_/CdS, with theoretical Ni contents of 1, 5, 10, 20, 25, 30, 35, 40 and 50 m/m%, defined as the molar mass of added Ni to CdS. The synthesis procedures for other transition metal-modified CdS (Co, Fe, Cu and Mn) were similar to that used for Ni_n_B@Ni-BO_x_/CdS. The concentration of transition metal precursor was controlled at 35 m/m% relative to CdS. The CdS-treated sample was obtained following the similar procedure as Ni_n_B@Ni-BO_x_/CdS, except without the addition of the Ni precursor.

Ni_n_B@Ni-BO_x_-Red./CdS was prepared by partially reducing Ni_n_B@Ni-BO_x_/CdS in a 5% H_2_/Ar atmosphere at 300°C for 1 h. Ni_n_B@Ni-BO_x_-Ox./CdS was prepared *via* the calcination of Ni_n_B@Ni-BO_x_/CdS in air at 300°C for 1 h. Ni_3_B was synthesized *via* a two-step chemical reduction. 1.0 g of nickel acetate was added to 50 mL of 10 mM sodium hydroxide aqueous solution under stirring, followed by the rapid addition of 5 mL of 1.2 M sodium borohydride aqueous solution. Following 10 min of stirring, the resulting precipitate was collected by centrifugation, washed with ultrapure water three times, and dried under vacuum at RT overnight. The obtained product was annealed in 5% H_2_/Ar atmosphere in a tube furnace at 300°C for 2 h with a ramp rate of 2°C min^–1^. To prepare Ni_3_(BO_3_)_2_, 0.71 g of nickel chloride, 0.20 g of sodium tetraborate and 0.09 g of sodium chloride were mixed and ground for 30 min. The resulting mixture was annealed in air at 800°C for 2 h. Following cooling to RT, the product was washed three times with ultrapure water and dried at 60°C. Ni_3_B/CdS and Ni_3_(BO_3_)_2_/CdS were fabricated *via* a self-assembly method. A selected amount of Ni_3_B ethanol dispersion was mixed with 50 mg of as-synthesized CdS under sonication for 30 min, followed by stirring for 12 h. Following drying at RT, Ni_3_B/CdS photocatalysts were obtained. The same method was applied to prepare Ni_3_(BO_3_)_2_/CdS, with Ni_3_B replaced by Ni_3_(BO_3_)_2_.

**Physicochemical characterizations.** X-ray diffraction (XRD) was conducted on a Rigaku MiniFlex 600 X-ray diffractometer using Cu Kα radiation. High-angle annular dark-field scanning transmission electron microscopy (HAADF-STEM) images, electron energy loss spectroscopy (EELS) analysis and energy dispersive spectroscopy spectra were determined on a FEI Titan Themis 80-200 (Thermo Fisher Scientific). X-ray absorption spectroscopy (XAS) measurements were performed on soft X-ray spectroscopy beamline of the Australian Synchrotron (ANSTO, Melbourne). Photo-irradiated X-ray photoelectron spectroscopy (XPS) spectra were collected on an ESCALAB 250Xi (Thermo Scientific) using a xenon lamp as light source. XPS and valence band (VB) spectra were determined on a Thermo Fisher Scientific K-ALPHA. Raman spectroscopy data were collected on a Via-Reflex spectrometer (Renishaw). UV-vis diffuse reflectance spectroscopy (UV-DRS) spectra were determined on a UV-vis 2600 spectrophotometer (Shimadzu). In situ diffuse reflectance infrared Fourier transform spectroscopy (DRIFTS) measurements were conducted on a Nicolet iS20 spectrometer equipped with a HgCdTe (MCT) detector cooled with liquid nitrogen. The photocatalysts were placed in a reactor, purged with Ar, and irradiated with a LED light. Fourier transform infrared spectroscopy spectra were determined on a Nicolet iS50 (Thermo Fisher). In situ Electron paramagnetic resonance spectra were determined on a Bruker EmxPlus using a xenon light. Steady photoluminescence spectra were determined on a FLS1000 spectrometer (Edinburgh). Solid-state nuclear magnetic resonance (NMR) spectra were determined on a Bruker Avance Neo 400WB. Temperature programmed desorption-mass spectrometry analysis was carried out on a Hiden DECRA. Inductively coupled plasma optical emission spectrometry analysis (Agilent 5110) was used to determine the Ni content in photocatalysts. An ASAP 2460 apparatus was used to determine the surface area.

**PET substrate pre-treatment.** 4 g of polyethylene terephthalate (PET) powder was dispersed in 80 mL of 2 M sodium hydroxide aqueous solution and heated at 60^o^C with stirring for 48 h. Following the reaction, the solution was filtered through 0.22 μm membranes to remove undissolved plastic particles. The filtrate was acidified to pH 3 with concentrated sulfuric acid, followed by membrane filtration to separate the terephthalic acid component. The resulting solution was neutralized with sodium hydroxide and subsequently filtered to obtain the PET substrate solution.

**Photocatalytic evaluation.** Photocatalytic performance was evaluated in a 160 mL custom-made reactor under ambient conditions. 10 mg of photocatalyst powder was ultrasonically dispersed in 10 mL of either 50 mM ethylene glycol (EG) aqueous solution or PET substrate solution. The photocatalyst suspension was purged with high-purity Ar for 20 min. Photocatalytic reactions were conducted under continuous stirring and irradiation using a 300 W xenon lamp (PLS-SXE 300, Beijing Perfectlight). At specific intervals, 100 µL of the headspace gas was sampled from the reactor and analyzed using Agilent 7890B gas chromatography. In parallel, an aliquot of the reaction solution was withdrawn and filtered to remove any photocatalyst. Quantification of products in each aliquot was performed using high-performance liquid chromatography (HPLC) (Thermo Scientific RefractoMax 520) and NMR (Agilent). For the product extraction, several batches of long-term reaction solutions were combined and filtered to remove photocatalyst particles. The filtrate was subsequently concentrated under reduced pressure or Ar purging to enrich the glycolaldehyde. A cold antisolvent mixture was then added with stirring, and the mixture was kept at -18°C for an extended period to induce crystal precipitation. The resulting crystals were collected by filtration and dried at low temperature.

The generation of the various products was determined using the following equation:

$Products generation=\frac{n_{\mathrm{products}}}{m_{photocatalysts or substrates}\times t}$

n_products_: the amount of products formed (μmol), m_photocatalysts_: the mass of photocatalysts used (g_cat_), m_substrates_: the mass of substrates used (g_sub_), t: the reaction time (h).

**Figure S1.** (a) HAADF-STEM image of Ni_n_B@Ni-BO_x_/CdS, with enlarged views of (b) the CdS component and (c) the Ni_n_B@Ni-BO_x_ component.

**Figure S2.** XRD patterns of CdS and Ni_n_B@Ni-BO_x_/CdS.

**Figure S3.** EELS mappings for Ni_n_B@Ni-BO_x_/CdS.

**Figure S4.** XRD patterns of (a) Ni_3_B and (b) Ni_3_(BO_3_)_2_. HAADF-STEM images of (c) Ni_3_B/CdS and (d) Ni_3_(BO_3_)_2_/CdS.

**Figure S5.** HAADF-STEM images of (a) Ni_n_B@Ni-BO_x_/CdS and (b) Ni_3_B/CdS. (c) Ni L-edge, (d) B K-edge and (e) O K-edge EELS spectra of Ni_n_B@Ni-BO_x_/CdS and Ni_3_B/CdS.

**Figure S6.** HAADF-STEM images of (a) Ni_n_B@Ni-BO_x_/CdS and (b) Ni_3_(BO_3_)_2_/CdS. (c) Ni 2p XPS spectra of Ni_n_B@Ni-BO_x_/CdS and Ni_3_(BO_3_)_2_/CdS. (d) B K-edge and (e) O K-edge EELS spectra of Ni_n_B@Ni-BO_x_/CdS and Ni_3_(BO_3_)_2_/CdS.

**Figure S7.** Photoreforming of EG substrate for pristine CdS, treated CdS and Ni_n_B@Ni-BO_x_/CdS, along with control experiments.

**Figure S8.** HPLC spectra for the reaction solution (a) before and (b) following the photocatalytic reaction on Ni_n_B@Ni-BO_x_/CdS. ^1^H NMR spectra for the reaction solution (c) before and (d) following the photocatalytic reaction on Ni_n_B@Ni-BO_x_/CdS.

**Figure S9.** Calibration curves for HPLC analysis.

**Figure S10.** Generation of products on Ni_n_B@Ni-BO_x_/CdS and differing transition metal-modified CdS following 5 h of EG photoreforming.

**Figure S11.** (a) HAADF-STEM image, (b) EELS mappings, (c) Ni 2p and (d) B 1s XPS spectra for Ni_n_B@Ni-BO_x_/CdS post reaction.

**Figure S12.** Raman spectra for prepared and commercial glycolaldehyde in dimer form.

**Figure S13.** UV-DRS for CdS and Ni_n_B@Ni-BO_x_/CdS.

**Figure S14.** (a) B 1s and (b) Cd 3d XPS spectra of Ni_n_B@Ni-BO_x_/CdS in dark and under illumination.

**Figure S15.** (a) HRTEM image of Ni_n_B@Ni-BO_x_-Red./CdS. (b) HAADF-STEM image for a selected area of Ni_n_B@Ni-BO_x_-Red./CdS. (c) Atomic percent composition based on the EELS line scan analysis in (b). (d) Ni 2p and (e) B 1s XPS spectra of Ni_n_B@Ni-BO_x_-Red./CdS.

**Figure S16.** (a) HRTEM image of Ni_n_B@Ni-BO_x_-Ox./CdS. (b) HAADF-STEM image for a selected area of Ni_n_B@Ni-BO_x_-Ox./CdS. (c) Atomic percent composition based on the EELS line scan analysis in (b). (d) Ni 2p XPS spectrum of Ni_n_B@Ni-BO_x_-Ox./CdS. (e) Ni L-edge XAS spectra of Ni_n_B@Ni-BO_x_-Red./CdS, Ni_n_B@Ni-BO_x_-Ox./CdS and Ni_n_B@Ni-BO_x_/CdS.

**Figure S17.** XPS VB spectra of (a) CdS and (b) Ni_n_B@Ni-BO_x_/CdS.

**Figure S18.** In situ DRIFTS spectra of control experiments on Ni_n_B@Ni-BO_x_/CdS.

**Figure S19.** DRIFTS spectra for differing concentrations of (a) EG, (b) glycolaldehyde and (c) acetic acid on Ni_n_B@Ni-BO_x_/CdS. (d) Enlarged view of the CO signal in the DRIFTS spectra shown in Figure 5a. (e) In situ C 1s XPS spectra of Ni_n_B@Ni-BO_x_/CdS with EG substrate and illumination.

**Figure S20.** In situ DRIFTS spectra for EG substrate conversion on Ni_3_B/CdS under light irradiation.

**Figure S21.** DRIFTS spectra for glycolaldehyde adsorption in dark and its photo-induced conversion on Ni_n_B@Ni-BO_x_/CdS.

**Table S1.** Selected physicochemical properties for CdS and Ni_n_B@Ni-BO_x_/CdS.

| **Sample** | **Theoretical Ni content**  **(m/m%)** | **Actual Ni content**  **(m/m%)** | **Surface area**  **(m^2^ g^−1^)** |
| --- | --- | --- | --- |
| CdS | N/A | N/A | 31.8 |
| Ni_n_B@Ni-BO_x_/CdS | 35 | 11.4 | 45.7 |

**Table S2.** Glycolaldehyde yield on CdS and Ni_n_B@Ni-BO_x_/CdS with varying Ni contents following 5 h of EG photoreforming.

| **Sample** | **Ni content**  **(m/m%)** | **Glycolaldehyde yield**  **(μmol)** |
| --- | --- | --- |
| CdS | 0 | 2.5 |
| Ni_n_B@Ni-BO_x_/CdS | 1 | 8.5 |
|  | 5 | 14.2 |
|  | 10 | 23.9 |
|  | 20 | 38.1 |
|  | 25 | 42.2 |
|  | 30 | 44.3 |
|  | 35 | 53.4 |
|  | 40 | 49.8 |
|  | 50 | 46.5 |

**Table S3.** Photocatalytic performance for selected state-of-the-art plastic reforming.

| **Photocatalyst** | **Main product(s)** | **Generation**  **(μmol g_cat_^−1^ h^−1^)** | **Generation**  **(μmol g_sub_^−1^ h^−1^)** | **Substrate** | **Condition** | **Reference** |
| --- | --- | --- | --- | --- | --- | --- |
| Ni_n_B@Ni-BO_x_/CdS | Glycolaldehyde | 1068.3 | 344.5 | EG | 300 W Xenon lamp | This work |
|  | Syngas | 3232.2 | 1042.7 |  |  |  |
| Ni_n_B@Ni-BO_x_/CdS | Glycolaldehyde | 613.3 | 13.6 | PET | 300 W Xenon lamp | This work |
|  | Syngas | 1821 | 40.5 |  |  |  |
| TiO_2_\|Pt | Pentanal | 100 | 8 | PCL | AM 1.5G | ^[1]^ |
|  | Hydrogen | 553 | 44.8 |  |  |  |
| Nb_2_O_5_ | Acetic acid | 0.7 | 0.1 | PVC | AM 1.5G | ^[2]^ |
| Hybrid-TiO_2_ | Ethanol | 16.06 | 5.35 | PE | AM 1.5G | ^[3]^ |
| Pd_1_Cu_0.4_-TiO_2_ | Formate | 4707 | 94.1 | PET | 300 W Xenon lamp | ^[4]^ |
| *M. b*-CDPCN | Methane | 12.6 | 0.8 | PLA | 395 nm LED | ^[5]^ |
| Pt-CdS | Pyruvic acid | 15978.3 | 39.9 | PLA | 300 W Xenon lamp (λ > 400 nm) | ^[6]^ |
|  | Hydrogen | 18683.3 | 46.7 |  |  |  |
| Co-Ga_2_O_3_ | Syngas | 750.7 | 375.4 | PP | AM 1.5G | ^[7]^ |

**References**

[1] S. Bhattacharjee, C. Guo, E. Lam, J. M. Holstein, M. Rangel Pereira, C. M. Pichler, C. Pornrungroj, M. Rahaman, T. Uekert, F. Hollfelder, E. Reisner, *J. Am. Chem. Soc.* **2023**, *145*, 20355-20364.

[2] X. Jiao, K. Zheng, Q. Chen, X. Li, Y. Li, W. Shao, J. Xu, J. Zhu, Y. Pan, Y. Sun, Y. Xie, *Angew. Chem. Int. Ed.* **2020**, *59*, 15497-15501.

[3] M. Jiang, J. Li, X. Wan, J. Qiu, T. Yao, W. Zhang, S. Ma, H. Tan, A. Han, C. Chen, G. Liu, *Nat. Commun.* **2025**, *16*, 4136.

[4] S. Zhang, B. Johannessen, B. Xia, X. Gao, K. Davey, J. Ran, S.-Z. Qiao, *J. Am. Chem. Soc.* **2024**, *146*, 32003-32012.

[5] J. Ye, Y. Chen, C. Gao, C. Wang, A. Hu, G. Dong, Z. Chen, S. Zhou, Y. Xiong, *Angew. Chem. Int. Ed.* **2022**, *61*, e202213244.

[6] Y. Miao, Y. Zhao, J. Gao, J. Wang, T. Zhang, *J. Am. Chem. Soc.* **2024**, *146*, 4842-4850.

[7] J. Xu, X. Jiao, K. Zheng, W. Shao, S. Zhu, X. Li, J. Zhu, Y. Pan, Y. Sun, Y. Xie, *Natl. Sci. Rev.* **2022**, *9*, nwac011.
